# The Mood and Resilience in Offspring (MARIO) project: a longitudinal cohort study among offspring of parents with and without a mood disorder

**DOI:** 10.1186/s12888-024-05555-z

**Published:** 2024-03-26

**Authors:** Annabel Vreeker, Melany Horsfall, Merijn Eikelenboom, Annemerle Beerthuizen, Veerle Bergink, Marco P. M. Boks, Catharina A. Hartman, Ricki de Koning, Max de Leeuw, Dominique F. Maciejewski, Brenda W. J. H. Penninx, Manon H. J. Hillegers

**Affiliations:** 1 Department of Child and Adolescent Psychiatry/Psychology, Erasmus University Medical Center, Rotterdam, The Netherlands; 2 Department of Psychology, Education and Child Studies, Erasmus University Rotterdam, Rotterdam, The Netherlands; 3 Department of Psychiatry, Amsterdam Public Health, Amsterdam UMC, Vrije Universiteit, Amsterdam, The Netherlands; 4 Department of Psychiatry, Erasmus University Medical Centre Rotterdam, Rotterdam, The Netherlands; 5 Department of Psychiatry, Icahn School of Medicine at Mount Sinai, New York, NY USA; 6 Department of Psychiatry, Brain Center University Medical Center Utrecht, University Utrecht, Utrecht, The Netherlands; 7 Interdisciplinary Center Psychopathology and Emotion Regulation (ICPE), Department of Psychiatry, University Medical Center Groningen, University of Groningen, Groningen, The Netherlands; 8 Department of Psychiatry, Amsterdam Public Health and Amsterdam Neuroscience, Amsterdam UMC, Vrije Universiteit, Amsterdam, The Netherlands; 9 Department of Psychiatry, Leiden University Medical Centre, Leiden, The Netherlands; 10 Mental Health Care Rivierduinen, Bipolar Disorder Outpatient Clinic, Leiden, The Netherlands; 11 Department of Developmental Psychology, Tilburg University, Tilburg, The Netherlands

**Keywords:** Mood disorder, Major depressive disorder, Bipolar disorder, Intergenerational transmission, Resilience

## Abstract

**Background:**

One of the most robust risk factors for developing a mood disorder is having a parent with a mood disorder. Unfortunately, mechanisms explaining the transmission of mood disorders from one generation to the next remain largely elusive. Since timely intervention is associated with a better outcome and prognosis, early detection of intergenerational transmission of mood disorders is of paramount importance. Here, we describe the design of the Mood and Resilience in Offspring (MARIO) cohort study in which we investigate: 1. differences in clinical, biological and environmental (e.g., psychosocial factors, substance use or stressful life events) risk and resilience factors in children of parents with and without mood disorders, and 2. mechanisms of intergenerational transmission of mood disorders via clinical, biological and environmental risk and resilience factors.

**Methods:**

MARIO is an observational, longitudinal cohort study that aims to include 450 offspring of parents with a mood disorder (uni- or bipolar mood disorders) and 100-150 offspring of parents without a mood disorder aged 10-25 years. Power analyses indicate that this sample size is sufficient to detect small to medium sized effects. Offspring are recruited via existing Dutch studies involving patients with a mood disorder and healthy controls, for which detailed clinical, environmental and biological data of the index-parent (i.e., the initially identified parent with or without a mood disorder) is available. Over a period of three years, four assessments will take place, in which extensive clinical, biological and environmental data and data on risk and resilience are collected through e.g., blood sampling, face-to-face interviews, online questionnaires, actigraphy and Experience Sampling Method assessment. For co-parents, information on demographics, mental disorder status and a DNA-sample are collected.

**Discussion:**

The MARIO cohort study is a large longitudinal cohort study among offspring of parents with and without mood disorders. A unique aspect is the collection of granular data on clinical, biological and environmental risk and resilience factors in offspring, in addition to available parental data on many similar factors. We aim to investigate the mechanisms underlying intergenerational transmission of mood disorders, which will ultimately lead to better outcomes for offspring at high familial risk.

**Supplementary Information:**

The online version contains supplementary material available at 10.1186/s12888-024-05555-z.

## Background

Between 15 and 28% of all children have a parent with a mental illness [1–3]. Mood disorders (i.e., major depressive disorder or bipolar disorder) are among the most common mental illnesses with lifetime prevalence rates in the general population ranging from 1–6% for bipolar disorder [4, 5] to approximately 10% for major depressive disorder [6]. In 2019, 280 million people worldwide were suffering from depression, among whom 23 million children and adolescents, and 40 million people were suffering from bipolar disorder [7]. Mood disorders severely impact the lives of patients as well as their family members, and are ranked among the leading causes of burden of disease by the World Health Organization [8]. Since a large proportion of patients with mood disorders have offspring, many children and adolescents are exposed to the stressors linked to parental mental illness (e.g., hospitalization, job loss, family conflict and other stressful life events). This, in addition to their increased genetic vulnerability [9], increases their risk of developing mood symptoms. Indeed, findings from prospective, cross-sectional and registry studies show that having a parent with a mood disorder is a strong risk factor for developing a mood disorder in their offspring [10]. For instance, offspring with a parent with bipolar disorder are 4 times more at risk of developing a mood disorder compared with children of healthy parents [11] and 50–65% of children with a parent with a mood disorder develop a mood disorder themselves before the age of 35 [12, 13]. Although clinical, biological and environmental factors are known to contribute to intergenerational transmission of mood disorders precise mechanisms underlying intergenerational transmission remain rather unclear [14].

Mood disorders often develop early in life, often during adolescence or young adulthood [15]. Experiencing mood symptoms during this sensitive developmental period can have long-lasting consequences for social, educational- and occupational functioning, underscoring the importance of early identification for timely treatment. In order to improve early identification of mood symptoms in youth at high familial risk, it is essential to understand which factors contribute to risk and resilience during the developmental period of adolescence and young adulthood. In the Mood and Resilience in Offspring (MARIO) project, we investigate the influence of clinical (i.e., type of parental disorder, illness severity, age at onset of the mood disorder, whether both parents are affected), biological and environmental (including psychosocial factors, substance use and stressful life events) factors explaining why some offspring develop a mood disorder while others do not. Here, we describe the rationale, objectives and methods of the study.

### Biological pathways for transgenerational transition of mood disorders

The development of mood disorders is partly influenced by biological factors. Family and twin studies show that mood disorders are moderately to highly heritable, with estimates around 37% for major depressive disorder [16] and 85% for bipolar disorder [17]. Genetic studies have shown that mood disorders are largely polygenic, which means that many genetic variants each have a small effect on the development of a mood disorder. Currently, 178 genetic loci have been associated with major depressive disorder [18]. For bipolar disorder, 64 genetic loci have been identified and it is expected that more genetic loci will be identified when sample sizes increase [19].

Few studies have investigated the contribution of genetic load to risk of developing psychopathology in offspring of parents with mood disorders. One study found that a higher genetic load for bipolar disorder was related to increased risk of transmission of bipolar disorder in offspring of parents with bipolar disorder [20] A study pooling data of 8 cohorts of offspring of parents with mood or psychotic disorders and controls (*N* = 1,884) found that genetic load for neuroticism and subjective well-being were, independent of family history, related to improved identification of risk of onset of major mood disorder and psychotic disorders [21]. These studies indicate a potential influence of genetic load on transmission of mood disorders over and above family history.

There is also evidence for epigenetic mechanisms to be associated with mood disorders [22, 23], but few studies have investigated this in offspring of parents with mood disorders. One study on 844 mother–child pairs from the Avon Longitudinal Study of Parents and Children (ALSPAC) found preliminary evidence for DNA methylation in cord blood of newborns with a mother with depression during pregnancy [24], but they did not replicate this finding in the Dutch longitudinal cohort study Generation R.

Other biological factors that are related to mood disorders involve stress system mechanisms, specifically the hypothalamic–pituitary–adrenal (HPA) axis [25, 26] and immune system [27]. However, results from high-risk offspring studies are equivocal. Whereas some studies showed HPA-axis hyperactivity measured in salivary cortisol in offspring of bipolar disorder patients [28] and daughters of mothers with a history of recurrent depression [29] as compared to offspring of parents without a mood disorder, others did not find a difference [30, 31]. A recent systematic review of 87 studies showed higher cortisol levels in offspring of parents with depressive disorders compared to controls [32]; it was also reported that only few studies have investigated cortisol levels in offspring of parents with bipolar disorder. In addition, several studies have shown signs of aberrant inflammation in offspring of parents with a mood disorder [30, 33–38]. The heterogeneous findings on the role of the stress and immune systems on mood disorders in offspring of parents with a mood disorder warrants further investigation.

### Environmental factors and mood disorders

Besides biological factors, environmental factors including psychosocial factors, substance use or stressful life events, also contribute to the risk of developing a mood disorder. Offspring of a parent with a mood disorder are raised in an environment that can pose more challenges (for instance because of hospitalization of the parent) as compared to offspring of parents without a mood disorder. Possibly as a result, offspring of parents with a mood disorder experience a higher load of stressful life events and more chronic stress exposure compared to children of parents without mood disorders [39, 40]. It is well-known that experiencing negative life events is an important contributor to increased risk of mood disorders [41, 42]. Importantly, offspring of parents with mood disorders are not only more often exposed to negative life events but may also be more susceptible to their effects compared to offspring of parents without a mood disorder; adolescents and young adults with a positive family history of depression experience more depressive symptoms or a greater risk of major depressive disorder after stressful life events compared to those without a positive family history of depression [43, 44]. However, contrasting findings are also reported. Findings from a longitudinal study show that although offspring of parents with bipolar disorder and major depressive disorder report more adverse environmental stressors than offspring of controls, these factors do not contribute to the transmission to (hypo)manic episodes [45]. In that study, traumatic experiences partially mediated the relationship between parental early onset major depressive disorder and elevated risk of major depressive disorder in offspring. In conclusion, although environmental factors are known to play an important role in the intergenerational transmission of mood disorders, it is still largely unclear how they influence intergenerational transmission precisely.

### Resilience

Despite the high emergence of mood disorders among offspring of parents with a mood disorder, many of these children will never develop mood symptoms and may be considered resilient. Resilience refers to the capacity of successful adaptation in the context of risk or threats [46–48]. It is a multi-system dynamic concept, indicating that it involves different systems, for instance at the individual, family, and community (e.g., school or neighborhood) level. Protective factors contribute to resilience of adolescents in different systems; positive coping styles (individual level), closeness with and support from parents (family level), and availability of support services (community level), all have an impact on the resilience of offspring [49, 50]. Although this does not apply to all offspring of parents with a mood disorder, studies suggest that protective factors are less present in this group of offspring. For example, studies suggest that offspring of parents with mood disorders show less optimal emotion-regulation and coping styles compared to offspring of parents of controls [51, 52] when exposed to stressors. Less optimal emotion-regulation and coping styles are related to the onset and recurrence of depression [53–56], and therefore important targets for prevention [57]. The present study was designed to investigate both risk and resilience factors in order to understand why psychopathology develops in part of our offspring sample while not in the remaining of the sample.

### Other high-risk offspring studies

In a systematic literature review, we identified 12 longitudinal studies in offspring of parents with major depressive disorder and bipolar disorder that have been carried out [58]. At baseline, these studies included between 129 and 701 (mean = 264) offspring. Notably, only 1 study included more than 500 offspring at baseline, and one-thirds did not include a control group of offspring with parents without a mood disorder, whilst a control group is important to identify risk and resilience factors that are specific to intergenerational transmission of psychopathology. In addition, we showed that in only 25% of the studies mental health problems in the co-parents, i.e., the partner of the parent with a mood disorder, were assessed directly, indicating that the majority of the studies had limited opportunities to examine the impact of both (biological) parents on risk of disease in the offspring. This is an important limitation, since it has been shown that psychiatric problems in the co-parent may further increase the risk of a mood disorder in offspring of psychiatric patients [13], whilst on the other hand, support from the co-parent can significantly reduce the risk for children to develop a mood disorder [59]. Furthermore, only few studies examined both children of parents with major depressive disorder and bipolar disorder (25%); examining both groups of children enables a cross-disorder approach, which is essential given the fact that bipolar disorder often starts with a depressive episode in offspring at risk for bipolar disorder [60]. Moreover, whereas many studies focused on risk factors of psychopathology, only few studies examined resilience factors in offspring. The present study was designed to investigate both risk and resilience factors in order to understand why psychopathology develops in part of our offspring sample while not in the remaining of the sample. Last, only 50% of the studies reported on biological factors in the parents and none of the studies investigated how genetic vulnerability in both parents was related to genetic vulnerability in their offspring. For a detailed description of earlier high-risk offspring studies, see [14].

### What is needed?

To improve our understanding of the intergenerational transmission of mood disorders, we urgently need longitudinal studies in which extensive data on clinical, biological and environmental factors is collected in a large sample of both high-risk and control offspring. In addition, clinical and biological data should be examined for both the index-parent and co-parent, as this will allow studying the unique contribution of risk through each parent. We believe that novel studies including these data will significantly contribute to existing longitudinal high-risk offspring studies and will provide important information on risk and resilience in high-risk offspring.

### Objectives of the current study

With a 3-year, 4-wave longitudinal, richly phenotyped observational study among offspring of parents with and without mood disorders, the Mood and Resilience in Offspring (MARIO) project aims to examine patterns of mood symptom development and resilience in high-risk offspring compared to control offspring. This study examines 1. differences in clinical, biological and environmental risk and resilience factors in children of parents with and without mood disorders, and 2. mechanisms of intergenerational transmission of mood disorders from both parents to children via clinical, biological and environmental risk and resilience factors.

The MARIO project provides a novel research infrastructure that adds to the existing offspring literature by various aspects. In particular, we will: 1. Create a new high-risk offspring longitudinal cohort study including a sample between 550 and 600 participants, 2. With not only offspring of patients (450 high-risk offspring) but also a control group of participants with parents without a mood disorder (100–150 controls), 3. Utilize detailed information on the index-parent which was already collected before the start of MARIO, including extensive data on psychopathology, genetics, immune markers, neuroimaging, and data on life events, and personality, 4. Obtain DNA and information on psychopathology of the co-parent, 5. Focus on both risk and resilience factors over time, 6. Examine both categorical and dimensional factors of psychopathology in offspring, allowing the study of a broad spectrum of symptoms, which is particularly important in young individuals who may not fulfill the criteria for a clinical diagnosis yet, 7. Investigate which factors contribute to potential sex differences in the development of depression, and 8. Study real-time daily emotions and behaviors through the Experience Sampling Method (ESM) to investigate whether mood dynamics are predictive of mood symptoms.

## Methods

### Design

MARIO is an observational, longitudinal cohort study in offspring of parents with a mood disorder and offspring of controls aged 10–25 years. Offspring were recruited from existing patient studies. Over a period of three years, 4 assessments will take place.

### Consortium

The MARIO project (www.mario-project.nl) is funded through a grant of the Netherlands Scientific Organization that stimulates research of psychiatric disorders (ZonMw, projectnumber 6361 00004). The MARIO Consortium exists of a large group of Dutch institutes, including 5 academic hospitals (Amsterdam University Medical Center, location VUmc, Erasmus University Medical Center, Leiden University Medical Center, University Medical Center Utrecht, University Medical Center Groningen), 4 universities (Vrije Universiteit Amsterdam, Leiden University, University Utrecht, Erasmus University), 6 mental health care institutions (Dimence, GGZ inGeest, GGZ Drenthe, GGZ Friesland, Lentis, GGZ Rivierduinen), 2 patient associations (PlusMinus, the Depression Society), 3 knowledge centers (Kenniscentrum Kinder- en Jeugdpsychiatrie, Kenniscentrum voor Bipolaire Stoornissen and Nederlands Kenniscentrum Angst, Dwang, Trauma en Depressie), a knowledge institute (Trimbos institute), experience centers (stichting me Me Mam, Augeo foundation), the association of Dutch municipalities (VNG) and health insurers (Zorgverzekeraars Nederland). In addition, we have a youth and adult panel consisting of offspring of parents with a mental illness and parents with a mood disorder. This panel was involved in the design of the study and was consulted during the study; for instance, input was used for the choice and wording of questionnaires, design of the MARIO app (which was used to measure daily mood and behavior), recruitment material and the website.

### Sample

The sample consists of offspring of parents with a mood disorder and offspring of parents without a mood disorder in the age of 10–25 years at baseline. We aim to include 550–600 participants with an equal gender distribution; at least 450 offspring of parents with a mood disorder and 100–150 offspring of controls. Participants are recruited from existing cohort studies; the Netherlands Study of Depression and Anxiety (NESDA) [61, 62], the Dutch Bipolar Cohort [63–65], Onderzoeksprogramma Peripartum Psychiatrie Erasmus MC Rotterdam *(Research Program Peripartum Psychiatry Erasmus MC Rotterdam;* OPPER) [66], IMAGE_AL [67], Dutch Bipolar and Schizophrenia Offspring Study (DBSOS) [68], MOod Treatment with Antidepressents or Running (MOTAR) [69], BIpolar Netherlands Cohort (BINCO) [70] and NormQuest [71]. These cohorts included either adult participants with and without mood disorders or offspring of parents with bipolar disorder and healthy controls. Offspring of parents with a main diagnosis that overlaps the mood disorder spectrum, i.e., anxiety disorder or psychotic disorder, were not excluded. Participants are excluded when the index-parent is not the biological parent and/or if the participant suffers from cognitive impairments based on information given by the parent. Parent diagnoses were confirmed through psychiatric interviews. Extensive clinical and environmental data as well as biological data from the index-parents or offspring are available within these cohorts. More information on these cohorts is provided in the Supplemental Material. Supplemental Table 1 shows an overview of the cohorts.

**Table 1:** Assessment of mental health in offspring

Domain	Instrument	Online	Age	Assessment
Psychopathology	Computerized Kiddie Schedule for Affective Disorders and Schizophrenia (K-SADS) – present and lifetime; DSM 5 version [72]	No	≥ 10	T0, T3
Depression/mania/ care use/care needs	Self-developed questionnaire ‘MARIO-check’, existing of: Depression items from (simplified) Patient Health Questionnaire (PHQ-9) [73], Mania items (questions based on K-SADS [72] and General Behavior Inventory (GBI) [74]; Items on psychotic symptoms, functioning, care use and need for care are self-developed	Yes	≥ 10	T0, T1, T2, T3
Psychopathology (dimensional)	Youth Self Report (YSR) [75] or Adult Self Report (ASR) [76]	Yes	YSR: 10–17 ASR: ≥18	T0, T1, T2, T3
Depression symptoms	Quick Inventory of Depressive Symptomatology-Self-Rated (QIDS SR) [77, 78]	Yes	≥ 13	T3
Obsessive–Compulsive Disorder (OCD) symptoms	Short OCD screener [79]	Yes	≥ 13	T0, T3

Online *yes indicates* online questionnaire that can be filled out at the test location or at home, *no* face-to-face assessment

### Procedure

**Fig. 1:**
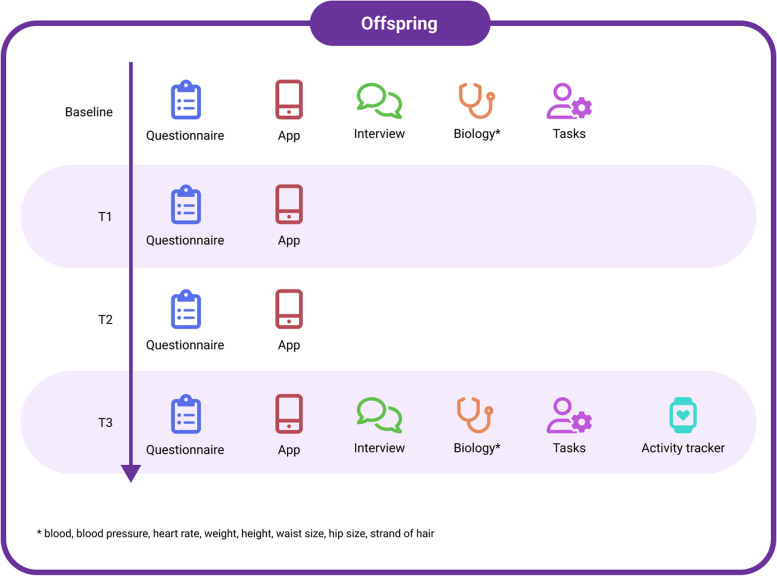
Overview of assessments in offspring (T0, T1, T2, T3)

**Fig. 2:**
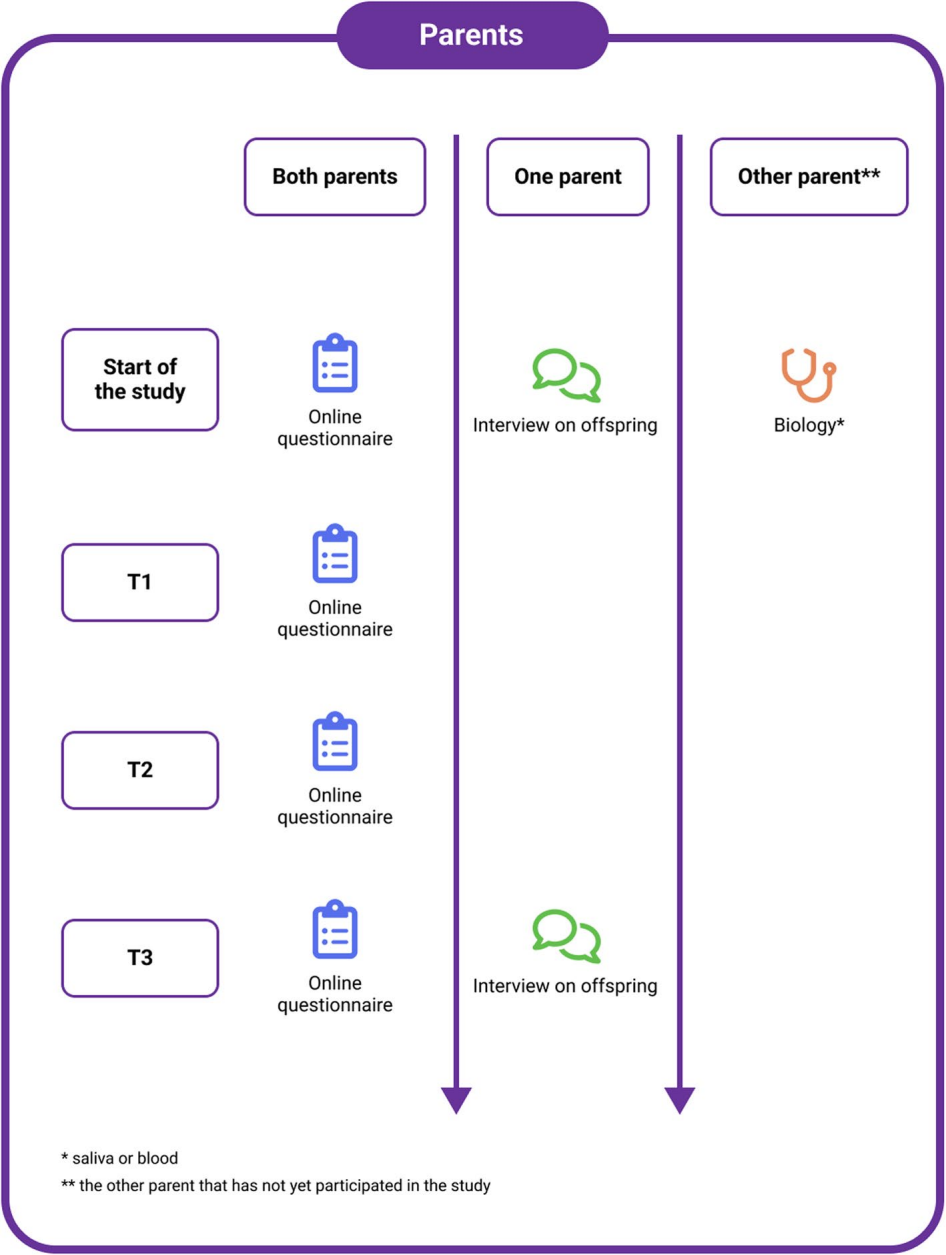
Overview of assessments parents (T0, T1, T2, T3)

During this 3-year longitudinal study, data are collected at 4 time points (baseline (T0), 1 year (T1), 2 years (T2) and 3 years (T3) after baseline). The procedures for offspring, parents and co-parents differ (see Figs. 1 and 2). Offspring are invited for both face-to-face and online assessments (see for instruments Tables 1 , 2, 3 and 4). Index-parents can be invited for collection of saliva or a blood sample, in case DNA is not available from the initial cohort studies. For their other phenotyping we rely on the initial cohorts that already collected detailed clinical and demographic data. Co-parents are invited for online assessments and saliva or blood collection. One of the parents is invited to join the face-to-face interview of their offspring in case the age of the offspring is below 18 years.

**Table 2:** Assessment of risk and resilience factors in offspring

Domain	Instrument	Online	Age	Assessment
**Sociodemographic**				
Demographics	Self-developed	No Yes	≥ 10	T0, T3 T1, T2
**Cognition**				
Cognition	Wechsler Intelligence Scale for Children fifth edition (WISC-V, subtests matrix reasoning and vocabulary) [80]; Wechsler Adult Intelligence Scale fourth edition (WAIS-IV, subtests matrix reasoning and vocabulary) [81]	No	WISC-V: 10–16 WAIS-IV: 17–28	T0, T3
**Psychological**				
Impairment	Impairment questions of fatigue scale from PROMIS [82]	Yes	≥ 10	T0
Resilience	Connor-Davidson Resilience Scale – 10 item version [83]	Yes	≥ 10	T3
Coping	Utrecht Coping List – Adolescent version (UCL-A) [84]	Yes	≥ 13	T0, T3
Self-esteem	Rosenberg Self-Esteem Scale [85]	Yes	≥ 13	T0, T3
Personality Big 5	Short version Next Big Five Inventory (BFI-2) [86]	Yes	≥ 13	T0, T3
Body image	Picture body image [87] and (questions body image and diet) from TRAILS [88]	Yes	≥ 10	T0, T1, T3, T3
**Social environment**				
Life events	Chronic difficulties and negative life events questionnaire, based on TRAILS [88]	Yes	≥ 10	T0, T1, T2, T3
Childhood trauma	Childhood Trauma Questionnaire (CTQ) [89]	No	≥ 10	T0, T3
Parental rearing	Parental bonding instrument (PBI) [90]	Yes	≥ 13	T0, T3
Parentification	Activity restrictions subscale of Young Carer of Parents Inventory (YCOPI) [91]	Yes	≥ 10	T0, T3
Sexting	Sexual health of adolescents in the Netherlands anno 2017 [92]	No	≥ 13	T0, T3
Media use	Frequency of use, based on the study Grumpy or Depressed [93]; Problematic internet use, based on Internet Addiction Test (IAT) [94]; Influence of social media, based on CBS item about influenceability of social media [95]	Yes	≥ 10	T0, T1, T2, T3 (short version at T3)
**Health and health behavior**				
Sexuality	Sexual health of adolescents [92]	Yes	≥ 13	T0, T3
Pubertal development	Physical Development Scale [96]	Yes	10–17	T0, T3
Sleep	Adapted School Sleep Habits Survey, [97]; chronotype from morningness-eveningness questionnaire [98]; sleep medication based on Nottingham Health Profile [99]	Yes	≥ 10	T0, T1, T2, T3
Sport	Questions used in the GenerationR study [100]	Yes	≥ 10	T0, T1, T2, T3
Medication	Medication questionnaire used in the study DBSOS [68]	Yes	≥ 10	T0, T3
Substance use	Substance Use based on the study TRAILS [88]	Yes	≥ 13	T0, T1, T2, T3
**Other**				
Evaluation of on-site assessment	Based on evaluation questionnaire used in the NESDA study [61, 62]	No	≥ 10	T0, T3

Online *yes* online questionnaire that can be filled out at the test location or at home, no face-to-face assessment, *DBSOS* Dutch Bipolar and Schizophrenia Offspring Study

**Table 3:** On-site physical assessment in offspring

Domain	Description	Assessment
Cardiovascular	Heart rate; systolic & diastolic blood pressure	T0, T3
Anthropometrics	Weight, height, waist circumference, hip circumference	T0, T3
Hypothalamic–pituitary–adrenal (HPA) axis activity	Cortisol in hair	T0, T3
Blood^a^	DNA, plasma, serum, hematology^b^ PBMC^b^	T0, T3

^a^In case a blood sample could not be collected; saliva was collected for DNA analysis

^b^In subsample of participants aged ≥ 16 years

**Table 4:** ESM, passive monitoring and actigraphy in offspring

**Domain**	**When**	**Instrument**	**Assessment**
**ESM (14 days; 5 × per day)**			
Sleep	Only in morning (1 × per day)	Self-developed, based on Grumpy or Depressed [93], GenerationR [100], NESDA [61, 62]	T0, T1, T2, T3
Location	5 × per day	Self-developed, based on Grumpy or Depressed [93], GenerationR [100], NESDA [61, 62]	T0, T1, T2, T3
Company	5 × per day	Based on Grumpy or Depressed [93], GenerationR [100], NESDA [61, 62]	T0, T1, T2, T3
Positive and negative affect	5 × per day	Items on positive and negative affect based on a previous studies [101], Grumpy or Depressed [93], GenerationR [100], NESDA [61, 62]	T0, T1, T2, T3
Coping	Only in the evening (1 × per day)	Based on Utrecht Coping List – Adolescent version (UCL-A) [84], adapted in Grumpy or Depressed [93]	T0, T1, T2, T3
Events	Only in the evening (1 × per day)	Based on a previous study on mood in adolescents [102]	T0, T1, T2, T3
Quality relationships	Only in the evening (1 × per day)	Based on Network of Relationships Inventory (NRI) [103]	T0, T1, T2, T3
Exercise	Only in the evening (1 × per day)	Self-developed, based on Grumpy or Depressed [93]	T0, T1, T2, T3
Substance use	Only in the evening (1 × per day)	Self-developed, based on Grumpy or Depressed [93], NESDA [61, 62], GenerationR [100]	T0, T1, T2, T3
**Passive monitoring (6 weeks)**^**a**^			
Location, calls & text, WIFI Access Point scans, screen states, real time app usage, ambient light, motion and step count	Continuously	Behapp smartphone app [104]	T3
**Actigraphy (14 days)**			
Physical activity	Continuously	Wrist-worn accelerometer GeneActiv [105]	T3
Sleep	In the evening/night for 2 weeks	Wrist-worn accelerometer GeneActiv [105]	T3
Activities of daily living	Continuously	Wrist-worn accelerometer GeneActiv [105]	T3

^a^Social behavior through passive mobile phone measures

### Offspring

#### Face-to-face assessments

At T0 and T3, face-to-face assessments take place. The face-to-face assessment takes approximately four hours to complete. Assessments are conducted at five Dutch research sites: Amsterdam University Medical Center, Erasmus University Medical Center, Leiden University Medical Center, University Medical Center Utrecht, and University Medical Center Groningen. After providing information on study participation and answering questions from participants, informed consent forms are signed. For participants under the age of 16, parents or the legal representatives of the participants co-sign the informed consent forms. The face-to-face assessment starts with a computer-assisted personal interview in which data on background, mental health, childhood trauma and sexual behavior are collected. Subsequently, biological measures (blood, a hair sample, blood pressure, height, weight, hip circumference and length) are collected. Two cognitive tasks are conducted to measure fluid intelligence (subtask Matrix Reasoning) and crystallized intelligence (subtask Vocabulary). The face-to-face assessment ends with an explanation about the online questionnaires and the ESM questionnaires. At T3, actigraphy measures are also administered.

#### Self-report questionnaire

At T0-T3 all offspring are asked to fill out online questionnaires on psychopathology, personality, activity, substance use, medication use, social media use, resilience, self-esteem and coping strategies. At T0 and T3 online questionnaires can be filled out at home or at the research site, depending on the preference of the participant. At T1 and T2, participants fill out the online questionnaire at home. It takes between 45 and 75 min to complete the online questionnaire, depending on age (i.e., younger participants fill out fewer questions than older participants). The online questionnaire can be filled out over several days to make it easier for children to complete.

#### Experience Sampling Method

At T0-T3 all offspring are asked to fill out ESM questionnaires on an application (the MARIO-app) on their mobile phone. For a period of 14 days, participants receive 5 micro-questionnaires per day on whereabouts, mood, coping strategies, activities, and substance use. At T3 participants are also asked to wear an actigraphy watch for 14 days (the same period in which the ESM questionnaires are filled out), which registers sleep and activity. At the same time, passive mobile phone data on social behavior is collected for a period of 6 weeks.

Participants receive a gift card for participating in the study and travel costs are reimbursed. Figure 1 shows the assessments for offspring.

#### Index-parent and/or co-parent report on offspring

When offspring are under the age of 18 years, one of the parents (the index-parent or the co-parent) is asked to join the face-to-face assessment of the offspring at T0 and T3 and participate in the psychiatric interview on their offspring. In addition, all index parents and co-parents are asked to fill out an online questionnaire about their participating offspring on mental health, exercise and education. This questionnaire can be filled out at home or at the test location, depending on the preference of the parent.

#### Index-parent and/or co-parent report on own health

All parents (biological and non-biological), who did not yet participate in one of the initial cohort studies are asked to provide information on demographics and mental health status through an online questionnaire. Index-parents and co-parents (only biological parents) for whom DNA is not available in the initial cohorts are asked to provide a blood or saliva sample for DNA analyses. Figure 2 shows the assessments for index-parents and co-parents.

### Measurement

#### Offspring

Questionnaires were chosen for their excellent psychometric properties and applicability for children and adolescents between 10–25 years. In addition, we have chosen instruments that overlap with instruments used in the Generation R study [100], TRacking Adolescents’ Individual Lives Survey (TRAILS) [88], NESDA [61, 62], Grumpy or Depressed [93], and DBSOS [68]); longitudinal studies in The Netherlands that follow children and adolescents (and/or parents). Tables 1, 2, 3 and 4 show a detailed overview of the instruments that were used at T0-T3 for offspring.

#### Index-parent and/or co-parent report on offspring

For participants < 18 years old, the index-parent or co-parent are invited at the on-site visits (T0 and T3) and interviewed on psychopathology of the child using the computerized K-SADS [72]. The visiting parent is also asked to fill out an evaluation form on the onsite visit. In addition, parents are asked to fill out online questionnaires on demographics of the child, the MARIO-check (consisting of the simplified PHQ-9 [73], mania questions based on the K-SADS [72] and General Behavior Inventory [74], and self-developed questions on functioning, care use and need for care), psychopathology of the child ((Child Behavior Checklist (CBCL; if child’s age < 18 years), Adult Behavior Checklist (ABCL; if child’s age ≥18 years.) [75, 76] and symptoms of autism spectrum disorder in the child (questions based on the K-SADS, [72]) at T0-T3.

#### Index-parent and/or co-parent report on own health

Index-parents or co-parents fill out online questionnaires at T0-T3 on the number and names of their children and (only for mothers) pregnancy and delivery (based on questions in TRAILS [88] and the expert opinion of one of the researchers (VB) in our consortium). When no DNA sample is available from the parent, we will also collect blood or saliva during the onsite visit (T0 or T3). For most index-parents, DNA is available through the initial cohorts. To obtain additional information on the co-parent (i.e., for index-parents detailed phenotype data is available through the initial cohorts), we ask co-parents to fill out online questionnaires at T0 on demographics, depression (using the Lifetime Depression Assessment Self-report (LIDAS) [106], symptoms of bipolar disorder (using the Mood Disorder Questionnaire (MDQ) [107] and care use (using the Trimbos and iMTA questionnaire on Costs associated with Psychiatric Illness) [108]. In addition, when the co-parent is not present during the onsite visit and no DNA sample is available, a saliva sample is collected through mail.

### Statistical analysis

Our aim is to build a large infrastructure with data that can be used for multiple research questions with multiple up-to-date statistical methods that are not described here (e.g., network analyses, machine learning approaches).

#### Objective 1: To examine differences in biological, clinical and environmental risk and resilience factors in children of parents with and without mood disorders

To analyze differences in symptoms, risk and resilience factors between high-risk and control offspring, we apply different statistical analyses, such as (non)linear latent growth curve (LGC) models, with parental psychopathology (mood disorder versus no mood disorder) as predictor and longitudinal data on clinical, biological and environmental risk and resilience factors as outcome. This will allow us to investigate whether the presence of a mood disorder of the parent can be related to both the levels (intercept) and change (slope) in symptoms, risk and resilience factors.

#### *Objective 2: To examine mechanisms of intergenerational transmission of mood disorders from both parents to children *via* clinical, biological and environmental risk and resilience factors*

We will examine which factors contribute to (differences in) the development of mood disorders and resilience. A statistical model that can be applied for this is growth mixture models (GMM). GMM are used to investigate subgroups in longitudinal developmental trajectories. These trajectories can be associated with biological and environmental factors using Structural Equation Modeling (SEM) to investigate differences in developmental trajectories (for instance to examine which factors are predictive of resilience in offspring of parents with a mood disorder). ESM data will be analyzed within subjects and between subjects to indicate whether changes in daily mood, measured over a period of two weeks, are predictive of the development of a mood episode. Daily mood profiles will be associated with transition to a mood episode using multilevel mixture models.

### Power calculation

We performed general power calculations to establish minimal detectable effect sizes (MDES) given the sample size of *n* = 550 for two basic situations. For the situation of a dichotomous outcome, the MDES is given in terms of Cohen's h. Using the 'pwr.2p2n.test' from R Package 'pwr', setting alpha = 0.05 and power = 1–beta = 0.80, we obtained Cohen's h = 0.268 as the MDES. For continuous outcome measures, the MDES is given in terms of Cohen's d. Assuming within subject correlation r = 0.5, number of measurements of m = 4, sd = 1 (because we are evaluating the standardized effect size of Cohen's d), using the formula of Twisk [109] and setting alpha = 0.05 and power = 1–beta = 0.80, we obtained Cohen's d = 0.218 as the MDES. In conclusion, a sample size of 550 children (450 high-risk offspring and 100 control offspring) is sufficient to find small- to medium-sized effects for our research questions.

### Data management

During the study, personal data (such as contact information, demographic variables and information concerning inclusion) is stored in an administrative/ electronic database at the secure server of the participating university medical center. Databases are only accessible by MARIO staff members. A six-number participant ID-number is created for every participant in the study to link the participant to the research data. Identifiable information will be kept separate from the collected research data. Only the local research staff at the different university centers have access to the key that connects the ID number to a person. Moreover, for biological data, laboratory personnel, biobank coordinators and researchers from the study will have access to the raw and processed biological data.

A Computer Assisted Personal Interview is used to collect interview data (locally on network drive or laptop). The online questionnaires are collected with an online data collection tool. A processor agreement is present at Amsterdam UMC for this. IP addresses are not collected from the online questionnaires. The MARIO App data is stored securely at a DMZ server at Erasmus MC. The data from the diagnostic interview (K-SADS) is stored on a server in the United States. In order to conform to the European privacy standards, a European Standard Contractual Clauses and a Data Processor Agreement is signed with the party that developed the online version of the K-SADS. Furthermore, we have added a section in the informed consent forms regarding storing of the data outside of The Netherlands as advised by the Privacy officer of the Erasmus MC. Researchers can only receive data—without privacy-sensitive data—from data management if the Principal Investigators have approved the analysis plan.

Pseudonymized data will be used for all statistical analyses. A structured protocol will be developed for data delivery, aggregation and integration of all data collected at different sites. These data are centrally cleaned by the data management team and delivered to researchers via safe data transfer methods. Quality control will be executed (out of range analysis, cross validation of variables, completeness of data) and a data dictionary will be developed for issuing of data. Personal and study data will be stored for 15 years after the study has ended and the personal data will be destroyed after this time period.

### Staff training and supervision

Assessments are administered by PhD students, research assistants or master students in the field of (mental) health. Research assistants and PhD students receive extensive training in conducting the T0 and T3 assessment according to the protocol. This training consists of two days of explaining and practicing the instruments that are used in the study, observing assessments conducted by an experienced interviewer and conducting assessments under supervision of an experienced interviewer. In order to maintain adherence to protocol and monitor data quality, supervision sessions take place every two weeks to discuss complex cases and reach consensus. Clinicians are available for consultation, for instance when consensus is not reached or in the event a participant reports serious mental health issues. Furthermore, local and central fieldwork meetings are organized to discuss practical issues related to the execution of the study. Protocols are in place for handling suicidality and suspicions of child abuse or neglect.

### Timeline and follow-up assessments

Recruitment for the MARIO cohort study started in November 2019 and will finish in the spring of 2024, which has been delayed due to the COVID-19 pandemic. The T1-assessment started in November 2020 followed by the T2-assessment in November 2021. The T3-assessment has started in August 2023. It is expected that T3 will end in the spring of 2027.

## Discussion

The MARIO study will be one of the largest longitudinal studies among offspring of parents with mood disorders worldwide. By examining extensive data on clinical, biological and environmental factors and data on risk and resilience in offspring of parents with a mood disorder, controls and their parents, our study aims to contribute to a better understanding of the mechanisms underlying intergenerational transmission of mood disorders, which will lead to improved identification of mood symptoms.

We have experienced delays in data collection (i.e., according to the original planning the baseline assessment should have already ended in 2021), mainly because of security regulations (i.e., the use of online assessments such as the ESM application and K-SADS online interview was carefully assessed by the Erasmus MC security officers), difficulties in recruitment and related to COVID-19 (i.e., research sites were closed for visits for several months, some participants were hesitant to come for an onsite visit because of infection risk, there were strict regulations in terms of symptoms of participants and interviewers which resulted in rescheduled visits).

The MARIO study will yield a new infrastructure for collaboration with other consortia and studies on high-risk offspring. Since we carefully selected instruments that are used in other longitudinal studies in The Netherlands, the MARIO study provides an excellent opportunity to compare youth at-risk of mood disorders with youth from the general population, which will result in a better understanding of the development of psychopathology in offspring of parents with a mood disorder.

Knowledge resulting from the MARIO study will improve early identification of mood disorders in offspring at high risk of developing a mood disorder. Early identification of mood disorders will facilitate early intervention and treatment, which may ultimately result in reduced treatment delays and improved outcomes for patients. The MARIO longitudinal cohort study is part of the broader MARIO consortium. In the MARIO study, we will, in two separate studies, further investigate the validity of an online tool to improve early identification of mood symptoms (MARIO screening study) and the efficacy of an online intervention platform (MARIO intervention study) [14, 110]. It is our ultimate goal to improve identification and early intervention for offspring at familial high risk, to reduce mental health problems and improve outcomes.

### Supplementary Information

**Additional file 1.**

## Data Availability

The datasets used and/or analyzed during the current study will be available from the corresponding author upon reasonable request.

## Abbreviations

ABCL: Adult Behavior Checklist
ASR: Adult Self Report
BFI: Big Five Inventory
BINCO: Bipolar Netherlands Cohort
CBCL: Child Behavior Checklist
CBS: Centraal Bureau voor de Statistiek (Central Bureau for Statistics)
CTQ: Childhood Trauma Questionnaire
DBSOS: Dutch Bipolar and Schizophrenia Offspring Study
DMZ: Demilitarized zone
DNA: Deoxyribonucleic acid
DSM 5: Diagnostic and Statistical Manual of Mental Disorders 5th edition
ESM: Experience Sampling Method
GBI: General Behavior Inventory
GGZ: Geestelijke Gezondheidszorg (Mental Health Care)
GMM: Growth Mixture Models
HPA-axis: Hypothalamic–pituitary–Adrenal axis
IAT: Internet Addiction Test
ID: Identification
iMTA: Institute for Medical Technology Assessment
IP: Internet Protocol
K-SADS: Kiddie Schedule for Affective Disorders and Schizophrenia
LGC: Latent Grow Curve
LIDAS: Lifetime Depression Assessment Self Report
MARIO: Mood and Resilience in Offspring
MDES: Minimal Detectable Effect Sizes
MDQ: Mood Disorder Questionnaire
MOTAR: MOod Treatment with Antidepressants or Running
NESDA: Netherlands Study of Depression and Anxiety
NRI: Network of Relationships Inventory
OCD: Obsessive compulsive disorder
OPPER: Onderzoeksprogramma Peripartum Psychiatrie Erasmus MC Rotterdam (Research Program Peripartum Psychiatry Erasmus MC Rotterdam)
PBI: Parental Bonding Instrument
PBMC: Peripheral Blood Mononuclear Cell
PhD: Doctor of Philosophy
PHQ: Patiënt Health Questionnaire
PROMIS: PRegnancy Outcomes and Insulin Sensitivity
QIDS-SR: Quick Inventory of Depressive Symptomatology-Self Rated
SEM: Structural Equation Modeling
TRAILS: Tracking Adolescents’ Individual Lives Survey
UCLA: Utrecht Coping List-Adolescent
VNG: Vereniging van Nederlandse Gemeenten (Association of Dutch Municipalities)
VUmc: Vrije Universiteit medisch centrum (Free University medical center)
WAIS: Wechsler Adult Intelligence Scale
WHO: World Health Organization
WISC: Wechsler Intelligence Scale for Children
YCOPI: Young Carer of Parents Inventory
YSR: Youth Self Report
ZonMw: Zorg Onderzoek Nederland Medische Wetenschappen (Care Research Netherlands Medical Sciences)
