# Supplementary material for: The Mood and Resilience in Offspring (MARIO) project: a longitudinal cohort study among offspring of parents with and without a mood disorder
Source: BMC Psychiatry. 2024 Mar 26;24:227. doi: 10.1186/s12888-024-05555-z (PMC10967130; doi:10.1186/s12888-024-05555-z)
Supplement: Supplementary file 1 — Additional file 1. [file 12888_2024_5555_MOESM1_ESM.docx]

**Supplemental Table 1. Overview of cohorts**

| **Cohort** | **Description** |
| --- | --- |
| NESDA | Longitudinal cohort study (13-year follow-up) in patients with anxiety and depression and healthy controls^1,2^ |
| The Dutch Bipolar Cohort | Cross-sectional cohort study in patients with bipolar disorder and healthy^4,5,6^ |
| OPPER | Longitudinal cohort study (4-year follow-up) in patients with postpartum depression and postpartum psychosis and healthy controls^9^ |
| IMAGE_AL | Cross-sectional cohort study in children of parents with bipolar disorder and postpartum depression^11^ |
| DBSOS | Longitudinal cohort study (3-year follow-up) in children of parents with bipolar disorder and healthy controls^12^ |
| MOTAR | Randomized controlled trial about the effects of running therapy vs. antidepressants in patients with major depressive disorder and healthy controls^16^ |
| BINCO | Longitudinal cohort study in patients with bipolar disorder^17^ |
| NormQuest | Cross-sectional cohort study in healthy controls^18^ |

*NESDA = the Netherlands Study of Depression and Anxiety; OPPER=Onderzoeksprogramma Peripartum Psychiatrie Erasmus MC Rotterdam (Research program peripartum psychiatry Erasmus MC Rotterdam), DBSO=the Dutch Bipolar Schizophrenia Offspring study; MOTAR = Mood Treatment with Antidepressants or Running; BINCO=Bipolar Netherlands Cohort*

**Supplemental Material**

**The Netherlands Study of Depression and Anxiety (NESDA)**

NESDA is an ongoing naturalistic, longitudinal cohort study in patients with anxiety and depression and healthy controls^1,2^. Patients are recruited through different health care settings (community, primary care and specialized mental health care). Anxiety and Mood disorder Diagnoses were confirmed using the Composite Interview Diagnostic Instrument (CIDI)^3^. Healthy controls were classified based on the absence of any CIDI-based lifetime psychiatric disorder.

**The Dutch Bipolar Cohort**

The Dutch Bipolar Cohort is a cross-sectional study that included patients with bipolar disorder, their relatives and controls from 2011-2015^4,5,6^. Patients were inpatients and outpatients recruited through Dutch psychiatric hospitals and institutions, the Dutch patient association, pharmacies and advertisements. The diagnosis of patients was assessed using the Structured Clinical Interview for DSM-IV (SCID-I)^7^. Controls were included if they did not have a diagnosis of bipolar disorder or a psychotic disorder as assessed using the Mini-International Neuropsychiatric Interview (MINI)^8^, nor did their first-degree relatives have a diagnosis of bipolar disorder or psychotic disorder according to self-report of the control. Controls were recruited through advertisement and among individuals who previously participated in scientific studies and agreed to be contacted for new research.

**Onderzoeksprogramma Peripartum Psychiatrie Erasmus MC Rotterdam *(Research Program Peripartum Psychiatry Erasmus MC Rotterdam)* (OPPER)**

OPPER is a longitudinal cohort study in women with postpartum depression and postpartum psychosis and healthy controls^9^. The case sample consisted of inpatients admitted to the Mother-Baby Inpatient Unit (MBU) of the department of Psychiatry of the Erasmus Medical Center, which treats patients with severe mental health problems postpartum (0-6 months).  Patients with a diagnosis of first-onset mania or psychosis during the postpartum period (aged 18-45 years) were included. Patient were diagnosed using the SCID-I/P. The control group existed of women from the general population who recently gave birth and did not have a self-reported psychiatric disorder, nor a postpartum depression as measured using the Edinburgh Postnatal Depression Scale^10^.

**IMAGE_AL**

IMAGE_AL is a cross-sectional study on the influence of lithium exposure during pregnancy on behavior, cognition and brain development of children of parents with bipolar disorder and postpartum depression^11^. The study included participants from 2017-2020. Diagnosis of the mother was based on structured screening of electronic medical files (obstetric and psychiatric) for all women who attended a perinatal mental health center at the Erasmus MC, LUMC or OLVG Amsterdam, and gave birth between 2003 and 2011.

**Dutch Bipolar and Schizophrenia Offspring study (DBSOS)**DBSOS is an ongoing prospective cohort study, that examines biological determinants (brain development and genetics), cognitive functioning, and environmental factors that are related to risk of psychopathology and resilience in offspring of patients with schizophrenia, bipolar disorder and controls^12^. Families with at least one first-degree relative or two second-degree relatives with schizophrenia or bipolar disorder are included. Participants were recruited via psychiatrists in The Netherlands, advertisements or hear-say. Control families were recruited via advertisements on schools and leisure clubs or hear-say. Diagnosis of the parents was confirmed using the SCID-I. In case this was not possible, diagnosis of the parent was determined using the Family Interview for Genetic Studies (FIGS)^13,14^. The mini Schedule for Clinical Assessment in Neuropsychiatry (mini-SCAN)^15^ interview was used for the co-parents and control parents. In case of reported psychopathology, a SCID-I interview was conducted. Controls were excluded when they, or a first-degree relative, had a severe mood or psychotic disorder.

**MOod Treatment with Antidepressants or Running (MOTAR)**

MOTAR is a randomized controlled trial that studies the effects of running therapy versus antidepressants in patients with major depressive disorder and healthy controls^16^.  Patients with a depressive disorder or anxiety disorder according to the CIDI (DSM-IV) who were enrolled at GGZ inGeest (a Dutch mental health care organization) between 2012 and 2019 were recruited for the study. Controls were recruited through advertisements and the study website. Controls, recruited through websites and social media, were included when they did not have a diagnosis of a psychiatric disorder according to the CIDI.

**Bipolar Netherlands Cohort (BINCO)**

BINCO is a longitudinal cohort study in patients who are recently diagnosed (<1 year) with bipolar disorder^17^ (Koenders et al., 2021). Patients were recruited through different mental health outpatient clinics in The Netherlands. The CIDI was used to confirm a diagnosis of bipolar disorder.

**NormQuest**

NormQuest is a cross-sectional cohort study in patients and healthy controls^18^. For the MARIO study, only healthy controls are selected. Healthy controls were recruited through random selection from registration systems of eight Dutch general practitioners. Participants were excluded when they received treatment for psychiatric disorders and/or alcohol or drug dependence within six months prior to the assessment. Controls were diagnosed using the MINI-Plus.

**References**

1. Penninx BW, Beekman AT, Smit JH, Zitman FG, Nolen WA, Spinhoven P, et al. The Netherlands Study of Depression and Anxiety (NESDA): rationale, objectives and methods. Int J Methods Psychiatr Res. 2008;17(3):121-40.
2. Penninx B, Eikelenboom M, Giltay EJ, van Hemert AM, Riese H, Schoevers RA, et al. Cohort profile of the longitudinal Netherlands Study of Depression and Anxiety (NESDA) on etiology, course and consequences of depressive and anxiety disorders. J Affect Disord. 2021;287:69-77.
3. Robins LN, Wing J, Wittchen HU, Helzer JE, Babor TF, Burke J, Farmer A, Jablenski A, Pickens R, Regier DA, Sartorius N, Towle LH. The Composite International Diagnostic Interview. Arch. Gen. Psychiatry. 1988;45: 1069-1077.
4. van Bergen AH, Verkooijen S, Vreeker A, Abramovic L, Hillegers MH, Spijker AT, et al. The characteristics of psychotic features in bipolar disorder. Psychol Med. 2019;49(12):2036-48.
5. Gilden J, Poels EMP, Lambrichts S, Vreeker A, Boks MPM, Ophoff RA, et al. Bipolar episodes after reproductive events in women with bipolar I disorder, A study of 919 pregnancies. J Affect Disord. 2021; 295:72-9.
6. van der Markt A, Klumpers U, Dols A, Korten N, Boks MP, Ophoff RA, et al. Accelerated brain aging as a biomarker for staging in bipolar disorder: an exploratory study. Psychol Med. 2023:1-10.
7. First MB, Spitzer RL, Gibbon M, Williams JBW. Structured Clinical Interview for DSM-IV Axis I Disorders-Patient Edition (SCID-I/P, version 2.0). Washington, D.C.: American Psychiatric Press, Inc. 1997.
8. Sheehan DV, Lecrubier Y, Sheehan KH, Amorim P, Janavs J, Weiller E, Hergueta T, Baker R, Dunbar, GC. The Mini-International Neuropsychiatric Interview (M.I.N.I.): the development and validation of a structured diagnostic psychiatric interview for DSM-IV and ICD-10. Journal of Clinical Psychiatry. 1998; 59(Suppl. 20): 22–33.
9. Bergink V, Lambregtse-van den Berg MP, Koorengevel KM, Kupka R, Kushner SA. First-onset psychosis occurring in the postpartum period: a prospective cohort study. J Clin Psychiatry. 2011;72(11):1531-7.
10. Cox JL, Holden JM, Sagovsky R. Detection of postnatal depression: development of the 10-item Edinburgh Postnatal Depression Scale. British Journal of Psychiatry. 1987; 150(6): 782-786.
11. Poels EMP, Kamperman AM, Bijma HH, Honig A, van Kamp IL, Kushner SA, et al. Brain development after intrauterine exposure to lithium: A magnetic resonance imaging study in school-age children. Bipolar Disord. 2023;25(3):181-90.
12. van Haren NE, Setiaman N, Koevoets MG, Baalbergen H, Kahn RS, Hillegers MH. Brain structure, IQ, and psychopathology in young offspring of patients with schizophrenia or bipolar disorder. European Psychiatry. 2020;63(1):e5.
13. Gershon ES, DeLisi LE, Hamovit J, et al. A controlled family study of chronic psychoses: schizophrenia and schizoaffective disorder. Arch Gen Psychiatry. 1988; 45(4): 328-336.
14. Maxwell ME. Family Interview for Genetic Studies (FIGS): A Manual for FIGS. Clinical Neurogenetics Branch, Intramural Research Program, National Institute of Mental Health. 1992.
15. Nienhuis FJ, van de Willige G, Rijnders CAT, de Jonge P, Wiersma D. Validity of a short clinical interview for psychiatric diagnosis: the mini-SCAN. Br J Psychiatry. 2010;196(1): 64-68.
16. Lever-van Milligen BA, Verhoeven JE, Schmaal L, van Velzen LS, Revesz D, Black CN, et al. The impact of depression and anxiety treatment on biological aging and metabolic stress: study protocol of the MOod treatment with antidepressants or running (MOTAR) study. BMC Psychiatry. 2019;19(1):425.
17. Koenders M, Mesbah R, Spijker A, Boere E, de Leeuw M, van Hemert B, et al. Effects of the COVID-19 pandemic in a preexisting longitudinal study of patients with recently diagnosed bipolar disorder: Indications for increases in manic symptoms. Brain Behav. 2021;11(11).
18. Schulte-van Maaren YW. NormQuest: reference values for ROM instruments and questionnaires. Leiden: Department of Psychiatry (Leiden University Medical Center); 2014.
